# Targeting myeloid villains in the treatment with immune checkpoint inhibitors in gastrointestinal cancer

**DOI:** 10.3389/fimmu.2022.1009701

**Published:** 2022-09-23

**Authors:** Chie Kudo-Saito, Narikazu Boku, Hidekazu Hirano, Hirokazu Shoji

**Affiliations:** ^1^ Department of Immune Medicine, National Cancer Center Research Institute, Tokyo, Japan; ^2^ Department of Oncology and General Medicine, Institute of Medical Science Hospital, Institute of Medical Science, University of Tokyo, Tokyo, Japan; ^3^ Department of Gastrointestinal Medical Oncology, National Cancer Center Hospital, Tokyo, Japan

**Keywords:** gastrointestinal cancer, immune checkpoint, myeloid cells, immunosuppression, inflammation, metastasis, treatment resistance

## Abstract

Despite the clinical outcomes being extremely limited, blocking immune inhibitory checkpoint pathways has been in the spotlight as a promising strategy for treating gastrointestinal cancer. However, a distinct strategy for the successful treatment is obviously needed in the clinical settings. Myeloid cells, such as neutrophils, macrophages, dendritic cells, and mast cells, are the majority of cellular components in the human immune system, but have received relatively less attention for the practical implementation than T cells and NK cells in cancer therapy because of concentration of the interest in development of the immune checkpoint blocking antibody inhibitors (ICIs). Abnormality of myeloid cells must impact on the entire host, including immune responses, stromagenesis, and cancer cells, leading to refractory cancer. This implies that elimination and reprogramming of the tumor-supportive myeloid villains may be a breakthrough to efficiently induce potent anti-tumor immunity in cancer patients. In this review, we provide an overview of current situation of the IC-blocking therapy of gastrointestinal cancer, including gastric, colorectal, and esophageal cancers. Also, we highlight the possible oncoimmunological components involved in the mechanisms underlying the resistance to the ICI therapy, particularly focusing on myeloid cells, including unique subsets expressing IC molecules. A deeper understanding of the molecular and cellular determinants may facilitate its practical implementation of targeting myeloid villains, and improve the clinical outcomes in the ICI therapy of gastrointestinal cancer.

## 1 Introduction

Blocking immune inhibitory checkpoint (IC) pathways, brakes on immune responses, has been in the spotlight as a promising strategy for treating diverse types of cancers, including gastrointestinal (GI) cancer, since the great therapeutic efficacies have been shown in the treatment with IC-blocking antibodies (ICIs) targeting cytotoxic T-lymphocyte-associated protein 4 (CTLA4) (ipilimumab and tremelimumab), programmed cell death protein 1 (PD1) (nivolumab, pembrolizumab, cemiplimab, and spartalizumab), and the PD1 ligand (PDL1) (atezolizumab, durvalumab, and avelumab), even in patients with advanced and metastatic cancer (1). The remarkable achievements have greatly contributed to changing the perception of cancer immunotherapy, and have led to development of a variety of immunotherapeutics, including blocking antibodies targeting other IC pathways or inflammatory pathways, peptide vaccines targeting tumor-associated antigens, and genetically engineered cellular products, for inducing anti-tumor responses in cancer patients (2, 3).

However, adverse events, including autoimmunity (4) and hyperprogression that is a rapid acceleration of the tumor growth and metastasis in patients shortly after treatment (5), are frequently observed in the treated patients. Also, the clinical response rate is relatively low, and most patients eventually show acquired resistance to the treatment even if responding in the beginning of the treatment (6). A reason may be that cancer cells affect numerous immunological components, including stromal cells, vascular cells, and immune cells, which in turn support cancer progression and metastasis. The reciprocal evolution increases heterogeneity and complexity of both tumor cells and the host immunity, leading to creation of refractory cancer (7).

To predict potential responses to anti-PD1/PDL1 therapy, biomarkers have been energetically investigated using advanced technology, and several biomarkers, including the PDL1 expression level as combined positive score (CPS) (8), the frequency of microsatellite instability (MSI) (9), or mutation burden (the number of non-synonymous single nucleotide variants) (10) in tumors have been identified so far. However, these are not necessarily correlated with clinical outcomes, and more precise and accurate biomarkers are still needed in clinical settings. To optimize the clinical efficacies of the ICI therapy, combination regimens with a variety of agents, such as small molecule inhibitors, other ICIs, and vaccines, have also been also evaluated in numerous clinical trials all over the world (3). However, the evaluation is still underway. A distinct strategy is obviously needed for the successful treatment of cancer.

Targeting myeloid cells, such as neutrophils, macrophages (Møs), dendritic cells (DCs), mast cells, may be a promising strategy for fundamentally changing such situation as a breakthrough in cancer immunotherapy. A reason is that myeloid cells are the majority of cellular components in the human immune system, and its abnormality may widely and negatively impact on the entire host, including tumor cells, stroma, and immunity, leading to treatment resistance, whereas the myeloid contents may vary within tumor microenvironment. In clinical settings, many studies have been demonstrated that local and systemic increase of myeloid cells is a poor prognostic marker in GI cancer as described later (11–13). Gut microbiome is known to regulate myelopoiesis, and its homeostasis and recruitment (14). Recent studies have revealed the crucial roles of gut microbiome in maintaining physiological conditions, including nutrient absorption and immune responses, and thus partly but significantly impacts on therapeutic efficacies induced by ICIs, such as anti-CTLA4 mAb (15), anti-PD1 mAb (16), and anti-PDL1 mAb (17). This suggests that elimination and reprogramming of the tumor-supportive myeloid cells facilitate induction of anti-tumor immune responses in the ICI therapy of GI cancer. However, targeting the myeloid villains is not yet practical in clinical settings, because a single/dominant marker that is exclusively and functionally expressed in the villain subsets, such as myeloid-derived suppressor cells (MDSCs), regulatory DCs (DCregs), and mesenchymal stromal/stem cells (MSCs), remain to be defined. To precisely distinguish the myeloid villain subsets is a priority issue for the practical application of myeloid-targeting therapy of cancer. Interestingly, accumulating evidence suggests that IC molecules, which are generally targeted on T cells and natural killer (NK) cells, are functionally expressed in myeloid cells expanded by cancer, and the unique sunsets promote tumor progression and metastasis directly and indirectly *via* inducing immune suppression and exhaustion leading to resistance to anti-PD1 therapy in mouse tumor models (18, 19). These suggest that the increased subsets are promising biomarkers to predict the potential unresponsiveness to anti-PD1 therapy. However, the clinical relevancy of targeting such myeloid subsets remains to be determined.

In this review, we provide an overview of background and current situation of the ICI therapy of GI cancer, and also highlight the oncoimmunological components involved in the mechanisms underlying the treatment resistance, particularly focusing on myeloid cells including the subsets expressing IC molecules. A deeper understanding of the molecular and cellular determinants would contribute to a practical implementation of targeting myeloid villains for improving the clinical effectiveness of the ICI therapy.

## 2 Background and current situation of the treatment of GI cancer

Development and the success of the ICI therapy surely changed the treatment paradigm for GI cancer in clinical settings (1). However, accumulating evidence suggests a limitation of the treatment due to innate or acquired resistance to the therapy in a majority of patients. To improve the clinical outcome, biomarkers have been explored to predict the potential responses to the ICI therapy, and numerous clinical trials have been conducted by combining a variety of agents for optimizing the therapeutic efficacy (**Table 1**). We firstly summarize the background and current situation of the treatments for GI cancers, including gastric cancer (GC), colorectal cancer (CRC), and esophageal cancer (EC), in clinical settings.

**Table 1:** Agents combined with anti-PD1/PDL1 mAbs in ongoing clinical trials (References).

Agents combined	Gastric cancer	Colorectal cancer	Esophageal cancer
Chemotherapeutics	Cisplatin/fluorouracil (20) Cisplatin/capecitabine (20)	Irinotecan/oxaliplatin/leucovorin/fluorouracil/bevacizumab (21) Temozolomide (22)	Fluorouracil/cisplatin (23)
Small molecule inhibitors	MKI Lenvatinib (24) MKI Regorafenib (25) HSP90 inhibitor TAS-116 (26) MMP9 inhibitor Andecaliximab (27, 28)	MKI Cobimetinib (29)	Lenvatinib (NCT04949256) Regorafenib (NCT04704154)
Immune checkpoint inhibitory mAbs	Anti-CTLA4 mAb (30) Anti-LAG3 mAb (31) Anti-TIGIT mAb (32)	Anti-CTLA4 mAb (33–35)	Anti-TIGIT mAb (NCT04732494, NCT04543617, NCT04540211)
Other therapeutics	Peptide vaccine OTSGC-A24 (36)	Anti-EGFR mAb cetuximab (37)	PD1-KO CAR-T targeting MUC1 (NCT03706326) CAR-T targeting EGFRvIII, DR5, NY-ESO-1 and Mesothelin (NCT03941626)

### 2.1 Gastric cancer

GC is the sixth most common type of cancer worldwide and ranks third among all causes of death due to malignant disease, while the age-adjusted incidence is decreasing globally (38). The reported risk factors are infection with Helicobacter pylori and Epstein-Barr virus (EBV), smoking, insufficient intake of vegetables and fruit, and alcohol consumption. GC types are histologically classified into two groups, diffuse and intestinal types, and the diffuse type is associated with peritoneal metastasis more frequently, but with hematological metastasis less frequently, as compared to the intestinal type (39). The Cancer Genome Atlas network divides GC into four molecular subtypes: EBV^+^ tumors (9%), MSI^+^ tumors (22%), tumors with genomic stability(20%), and tumors with chromosomal instability (50%) (40). Local GC can be cured by surgical resection with or without perioperative adjuvant chemotherapy, and systemic chemotherapy is the standard treatment of patients with advanced, unresectable, and recurrent GC (AGC) (41). Since late 1980’s and early 1990’s, combination of fluoropyrimidine (5-fluorouracil, capecitabine and S-1) and platinum (cisplatin and oxaliplatin) has been commonly and globally used. In late 1990’s, docetaxel, paclitaxel, and irinotecan were clinically developed, showing a survival benefit compared with the best supportive care as the second line treatment (42). Recently, trifluridine tipiracil prolonged survival as the third or later line treatment (43).

In 2000’s, a door of molecular targeted agents was opened for treating various kinds of malignant diseases. However, there are few options of the agents for treating AGC. For example, survival benefits of anti-HER2 monoclonal antibody (mAb) taruzuaumab in combination with fluoropyrimidine and platinum have been reported as the first-line treatment of HER2^+^ AGC patients (44), amplification or overexpression of HER2 gene are seen only in 10 - 20% of GC. Anti-angiogenic inhibitor ramucirumab combined with weekly paclitaxel also prolonged survival as the second-line treatment (45). Recently, trastuzumab deruxtecan, which is an anti-HER2 mAb conjugated with containing topoisomerase I inhibitor, showed a higher response rate and longer survival of HER2^+^ AGC patients as compared to the physician’s choice of chemotherapy as the third or later line treatment (46). However, the overall outcome has been low.

A rise of ICIs dramatically changed the situation. The ATTRACTION-2 study that is the first pivotal trial demonstrated a survival benefit of anti-PD1 mAb nivolumab as compared to placebo as the third or later line treatment of AGC (median survival 5.26 versus 4.14 months, hazard ratio [HR] = 0.63, P < 0.0001) (47). Recent phase III trials showed positive results as the first line treatment of AGC. For example, the Checkmate-649 study reported nivolumab plus oxaliplatin-based doublet chemotherapy prolonged overall survival (OS) in patients with CPS ≥ 5 or ≥ 1 tumors, and all randomized patients (48). The ATTRACTION-4 study conducted in Asian countries also reported the benefit of nivolumab therapy showing significantly longer progression-free survival (PFS) (49). Now, nivolumab has been approved for AGC as the first line treatment in many countries. Anti-PD1 mAb pembrolizumab has been additionally approved for MSI-H AGC as the second or later line treatment, while the incidence of MSI-H is only 5% in AGC (50).

Durable response is a strong point of the ICI therapy. For example, duration of response was as long as 9.5 months in AGC patients even as the third-line treatment with nivolumab in the ATTRACTION-2 study (51), 18.0 months in patients with CPS ≥ 1 tumors as the second-line treatment with pembrolizumab in the Keynote-061 study (52), and 13.7/19.3 months in patients with CPS ≥ 5/≥ 10 tumors, respectively, as the first-line treatment with pembrolizumab in the Keynote-062 study (20). The response durations are longer than cytotoxic agents in AGC. However, the clinical responses are low in the ICI therapy, and more than half of the patients showed progressive disease soon after treatment, suggesting innate and acquired resistance to the treatment (47). The KEYNOTE-061 study reported that pembrolizumab showed no significant survival benefit even in AGC patients with CPS ≥ 1 tumors as compared to weekly paclitaxel as the second-line treatment (52). The Javelin Gastric 300 trial reported that anti-PDL1 mAb avelumab showed slightly inferior survival as compared to the physician’s choice of chemotherapy as the third-line treatment (53). In addition, the ATTRACTION-2 study reported that 2- and 3-year PFS rates were only 3.8% and 2.4% in all patients receiving nivolumab as third or later line treatment of AGC (51). Also, the 2-year update analysis of the Keynote-061 study reported that disease progression was seen in 95.4% (377/395) of patients with CPS ≥ 1 tumors, 93.5% (174/186) of patients with CPS ≥ 5 tumors, and 89.8% (97/108) of patients with CPS ≥ 10 tumors as the second line treatment with pembrolizumab (54).

Therefore, biomarkers to predict the therapeutic efficacy have been explored in the ICI therapy, and some factors, including PDL1-CPS score, deficiency of mismatch repair (dMMR), and the frequency of MSI and tumor mutation burden (TMB), have been suggested as diagnostic biomarkers to guide the application of anti-PD1/PDL1 mAbs. PDL1 overexpression in tumor tissues is the first biomarker expected in the anti-PD1/PDL1 therapy. PDL1 overexpression is seen in 25 - 65% of GC patients, and several clinical studies have demonstrated that the high levels of PDL1 are associated with lymph node metastasis, late stage of the disease, and poor prognosis (55, 56). Then, pembrolizumab was approved by the FDA for selectively treating CPS ≥ 1 advanced GC or gastroesophageal junction adenocarcinoma based on the positive results of the KEYNOTE-059 study showing significantly higher response in patients with PDL1^+^ tumors as compared to patients with PDL1^-/low^ tumors (57). Another outstanding biomarker is genomic abnormality that is unable to maintain genomic integrity in tumor cells. The high frequency of MSI (MSI-H) and dMMR are observed in 8 - 37% of GC patients, and TMB is seen in about 11% of GC patients (58). Many clinical studies have demonstrated that the MSI-H/dMMR status is associated with significantly better response and survival outcome in the anti-PD1/PDL1 therapy (59).

However, the conclusions of the clinical significance are still controversial. For example, the Keynote-062 study reported that pembrolizumab monotherapy was not superior to chemotherapy in patients with CPS ≥ 1 tumors, although providing a clinically meaningful benefit in OS of patients with CPS ≥ 10 tumors, and combination of pembrolizumab plus chemotherapy (cisplatin and fluorouracil, or capecitabine) was not also superior to chemotherapy alone in OS of patients with CPS ≥ 1 or ≥ 10 tumors, suggesting the insufficiency of the CPS as a predictive biomarker (20). Thus, combination regimens with other agents, have been alternatively evaluated in many clinical trials for improving the efficacy of the ICI monotherapy of GC. In most cases, anti-PD1/PDL1 mAbs have been combined with other ICIs targeting another IC pathways, such as anti-CTLA4 mAb (30), anti-lymphocyte-activation gene 3 (LAG3) mAb (31), and anti-T cell immunoglobulin and ITIM domain (TIGIT) mAb (32), or small molecule inhibitors targeting the malignant properties of tumor cells (proliferation, differentiation, adhesion, apoptosis, and migration) and angiogenic signaling (60). For example, anti-angiogenic inhibitors, such as regorafenib and lenvatinib, have been clinically evaluated in combination with anti-PD1 therapy. Lenvatinib plus pembrolizumab showed a promising response rate of 66% in 29 patients as the first- or second-line treatment for AGC (24), and regorafenib plus nivolumab also showed a response rate of 44% in 25 AGC patients as the two or more lines of prior chemotherapy in the REGONIVO/EPOC1603 study (25). Now, the LEAP-5 study is underway for evaluation of the combination efficacy of pembrolizumab plus lenvatinib in various solid tumors, including AGC.

However, most clinical trials have shown no synergistic benefits of the combination. For example, no benefits were seen in AGC patients in a phase Ib trial using an inhibitor (TAS-116) of HSP90, which facilitates NLRP3 inflammasome activity during inflammatory responses, in combination with nivolumab (26). Also, no benefits were seen AGC patients in the randomized phase II trial using a matrix metalloproteinase 9 (MMP9) inhibitor andecaliximab in combination with nivolumab (27), although much better responses (5/10 = 50%) were seen in Japanese patients with GC or gastroesophageal junction adenocarcinoma in a phase 1b trial (28). Active immunotherapy has been also clinically evaluated in the treatment of GC. However, most trials have failed. For example, no objective response was observed in a phase I trial with OTSGC-A24 that is an HLA-A*24:02-binding cocktail peptide vaccine targeting multiple tumor antigens (FOXM1, DEPDC1, KIF20A, URLC10 and VEGFR1), although responses of cytotoxic CD8^+^ T cells (CTLs) were enhanced in 75% of AGC patients at 4 weeks after vaccination (36).

### 2.2 Colorectal cancer

CRC is the third most common primary tumor worldwide and ranks second in terms of mortality (38). Standard conventional treatments for CRC are surgery, chemotherapy and radiotherapy, and these treatments are combined depending on the localization and progression of the disease (61). Complete remission is often unachieved, and > 60% of stage II/III patients require further treatments with irradiation, chemotherapeutics, molecule targeting agents, and/or immunotherapeutics. As described in the GC section, ICI application dramatically changed the treatment paradigm for CRC. PDL1 is overexpressed in about 53% of CRC, but the level is rarely associated with clinical responses to the ICI therapy (62, 63). In contrast, the MSI-H/dMMR status is a strong biomarker to predict potential CRC responders to the ICI therapy. However, the frequency of MSI-H and dMMR varies across tumor types and stages, and the high frequency of the MSI-H/dMMR is observed only in 15 - 19% of CRC (64). The Keynote-177 study reported that pembrolizumab monotherapy showed significantly longer median PFS (16.5 vs. 8.2 months, HR = 0.60, P = 0.0002) than the standard-of-care chemotherapy as the first-line treatment of metastatic MSI-H/dMMR CRC (65). This result led to the FDA approval of pembrolizumab for the first-line treatment of patients with unresectable or metastatic MSI-H/dMMR CRC (66).

The accumulating evidence conversely suggests that the anti-PD1/PDL1 monotherapy is insufficient for treating the rest majority of CRC, microsatellite-stable and MMR-proficient tumors. Therefore, combination regimens with many other agents have been evaluated in numerous clinical trials. For example, the AtezoTRIBE study reported that atezolizumab and chemotherapy (irinotecan, oxaliplatin, leucovorin, fluorouracil, and bevacizumab) significantly prolonged PFS as compared to the chemo-control (21). However, the CheckMate 9X8 phase II/III trial reported at the GI Cancers Symposium 2022 that nivolumab in combination with the standard-of-care chemotherapy (5-fluorouracil, leucovorin, oxaliplatin, and bevacizumab) showed no synergistic effect on PFS in previously untreated patients with metastatic CRC. Molecular targeting small molecule inhibitors have been also combined with the ICIs. For example, Gomez-Roca et al. reported at ASCO 2021 that a multikinase inhibitor lenvatinib synergized with pembrolizumab in producing potent antitumor activity (objective response rate = 22%, and median PFS = 2.3 month) in patients with CPS ≥ 1 tumors in a nonrandomized phase II trial. Many other combination regimens have been now clinically developed: For example, a MAPK signaling inhibitor cobimetinib plus atezolizumab (29), anti-epidermal growth factor receptor (EGFR) mAb cetuximab plus anti-PDL1 mAb avelumab (37), and an alkylating agent temozolomide plus low-dose ipilimumab/nivolumab (22).

The most commonly combined agents are other ICIs targeting another IC pathway. The NICHE study reported that neoadjuvant treatment with a single dose of ipilimumab and two doses of nivolumab showed 100% pathological response in dMMR tumors, and 27% pathological response in MMR-proficient tumors of early-stage CRC patients within 4 weeks after treatment (33). The CheckMate-142 study reported that combination of nivolumab plus low-dose ipilimumab provided robust and durable clinical benefit as the first-line treatment of metastatic MSI-H/dMMR CRC, regardless of the PDL1 expression or the BRAF/RAS mutation status (34). Combination with anti-PDL1 durvalumab and anti-CTLA4 tremelimumab also provided better prognosis (2.5-month improvement of OS) in patients with advanced refractory CRC as compared to the best-supportive-care control in a phase II trial (35). Garralda et al. (abstract #3584) reported at ASCO 2021 that four patients presented partial response and one patient achieved complete response in the phase I first-in-human study using anti-LAG3 antibody MK4280 (favezelimab) and pembrolizumab for 89 patients with advanced microsatellite-stable CRC. The results encouraged the further development of MK4280, and the phase III trial is currently underway.

### 2.3 Esophageal cancer

EC is ranked as the seventh most common cancer, and is the sixth leading cause of cancer-related mortality in 2020 worldwide (38). EC is characterized by male dominance, geographic variation in incidence, and poor survival in the advanced stage, and is histologically divided into two major subtypes: esophageal squamous cell carcinoma (ESCC) that is the most common subtype (about 85% globally), and esophageal adenocarcinoma (EAC) (67). Profiles of genetic alterations differ between ESCC and EAC. Mutations in NFE2L2, MLL2, ZNF750, NOTCH1, and TGFβR2 are frequently observed in ESCC, but CDKN2A, ARID1A, SMAD4, and ERBB2 in EAC (68). Here, we mainly mention about advanced ESCC with high TMB but low frequency (1.08%) of MSI-H (69), since EAC is treated according to the strategy for GC.

Before the advent of ICIs, cytotoxic agents play crucial roles in the systemic chemotherapy for treating advanced ESCC, providing palliation of symptoms and prolongation of survival. Historically, fluorouracil-based or platinum-based chemotherapy are considered as the standard-of-care chemotherapy as the first line setting, and taxan agents (e.g., paclitaxel) as the second-line or later setting. Molecular targeting inhibitors, such as a small molecule EGFR inhibitor gefinitib (70) and anti-EGFR blocking mAb panitumumab (71), have been evaluated in phase III trials in advanced EC, while no clinical benefit has been shown. The rise of ICIs revolutionarily changed the treatment landscape of advanced EC, and the ICI therapy is now a standard treatment of pretreated patients with advanced ESCC. The ATTRACTION-3 study that is an international randomized phase III study reported that nivolumab provided significant better prognosis as compared to chemotherapy (docetaxel or paclitaxel) (median OS = 10.9 versus 8.5 months, HR = 0.79, P = 0.0264; 3-year OS rates = 15.3% versus 8.7%) in patients with ESCC refractory to fluoropyrimidine and platinum (23). Other phase III studies using another anti-PD1 mAbs, such as pembrolizumab (72), camrelizumab (73), tislelizumab (74), reproduced the anti-PD1 efficacy in the treatment of pretreated ESCC. Despite the great achievement, however, patients with advanced ESCC mostly experience disease progression after the treatment. Therefore, the response-predictive biomarkers and combination regimens to produce the synergistic effect have been explored for treating EC. However, the MSI/dMMR/TMB status is relatively low in EC (MSH-H in 5 - 10%, dMMR in 3 - 5%, and TMB in 2% of EAC and 0% of ESCC) (75), and no large-scale study has demonstrated the significance of the MSI/dMMR/TMB status in the ICI therapy of EC.

On the other hand, PDL1 expression has been considered as a useful biomarker to predict potential responses to the ICI therapy. PDL1 overexpression is observed in about 20% of EC patients, particularly with ESCC (76), and is significantly associated with lymph node metastasis, later disease stage, and poor prognosis (55). The CheckMate-648 study reported that combination of chemotherapy (fluorouracil and cisplatin) plus nivolumab provided significantly better prognosis (median OS = 15.4 versus 9.1 months, HR = 0.54, P < 0.001) as compared to chemotherapy alone in patients with unresectable advanced, recurrent, or metastatic previously untreated ESCC patients with CPS ≥ 1% tumors (23). In addition, combination of nivolumab plus ipilimumab provided significantly better prognosis (median OS = 13.7 versus 9.1 months, HR = 0.64, P = 0.001) as compared to chemotherapy alone in patients with PDL1^+^ tumors. Other phase III trials using another anti-PD1 mAbs, such as pembrolizumab (77), camrelizumab (78), sintilimab (79) and toripalimab (80), reproduced the significant anti-PD1 therapeutic efficacy compared to the chemotherapeutic efficacy in patients with advanced ESCC as the first-line settings.

Other agents, such as anti-angiogenic agents and other ICIs, have been clinically evaluated in combination with anti-PD1/PDL1 therapy. For example, there are two studies using small molecule multikinase inhibitors: regorafenib plus nivolumab in a phase II study (NCT04704154), and lenvatinib plus pembrolizumab in a phase III study (NCT04949256). Other ICIs targeting another IC pathway, including T-cell immunoglobulin domain and mucin domain 3 (TIM3), TIGIT, and LAG3, have been mostly combined in clinical trials. The high levels of TIM3 and TIGIT are associated with poor prognosis in ESCC (81), and LAG 3 is upregulated in CD8^+^ T cells and NKT cells in patients with ESCC (82). These new ICIs are currently under investigation in many clinical trials for EC. For example, the AdvanTIG-203 study is a phase II study using anti-PD1 mAb tislelizumab and anti-TIGIT mAb ociperlimab (NCT04732494). The SKYSCRAPER-07 is a phase III study using atezolizumab plus another anti-TIGIT mAb tiragolumab (NCT04543617). The SKYSCRAPER-08 is a phase III study using chemotherapy with paclitaxel and cisplatin in addition to the immunotherapy with atezolizumab plus tiragolumab (NCT04540211).

To overcome innate and acquired resistance to immunotherapy, cell products with genetically engineered T cells has been clinically developed in cancer therapy. Particularly, T cells transduced with chimeric receptors composed of intracellular domains of immunoreceptors (CD3ζ, CD28 and/or 4-1BB, etc.) and single chain variable domain fragments (scFv) of tumor antigen-specific mAbs, called “chimeric antigen receptor T-cell (CAR-T)”, have been clinically developed for treating cancer, including advanced EC. For example, a phase I/II study has evaluated the therapeutic efficacy of MUC1-targeting and PD1-knockout CAR-T cells (NCT03706326). Another phase I/II study has evaluated the therapeutic efficacy of CAR-T targeting multiple tumor antigens (EGFRvIII, DR5, NY-ESO-1 and Mesothelin) (NCT03941626). However, most trials are still underway.

## 3 Heterogeneity and complexity of the oncoimmunological network

Why is the immune system of cancer patients insensitive to the ICI therapy? A strong reason is enormous heterogeneity and complexity of the oncoimmunological network produced by the interplay between tumor cells and host immunity in cancer patients. Tumor-specific CTLs are generated and activated *via* the immune complexes composed of the T-cell receptor (TCR) and antigen peptide-loading major histocompatibility complex molecule I/II (MHC I/II) expressed on antigen-presenting cells (APCs), such as DCs, B cells, and Møs. Stable engagement with costimulatory molecules, including CD80, CD83, and CD86, is necessary for intensification of the TCR/MHC/peptide stimulatory signals to induce potent CTLs against cancer (83). However, this immune activation cascade is sometimes neglected and interfered by tumor cells. Firstly, tumor cells have an intrinsic potential to evade the immune attack by multiple ways. For example, tumor cells frequently express no or rare MHC I/II due to decrease or inactivation of an oncosuppressor TP53 (84). Also, tumor cells acquire high mobility and cancer stemness, including high self-renewability and anti-apoptotic dormancy contributing to treatment resistance, through a an evolutionarily conserved biological program “epithelial-to-mesenchymal transition (EMT)” in response to various stimuli within the tumor milieu (85). The EMT signaling through the RAS/ERK pathway upregulates PDL1 expression for braking the activation signaling pathways in anti-tumor effector cells by binding to PD1 (86).

**Figure 1:**
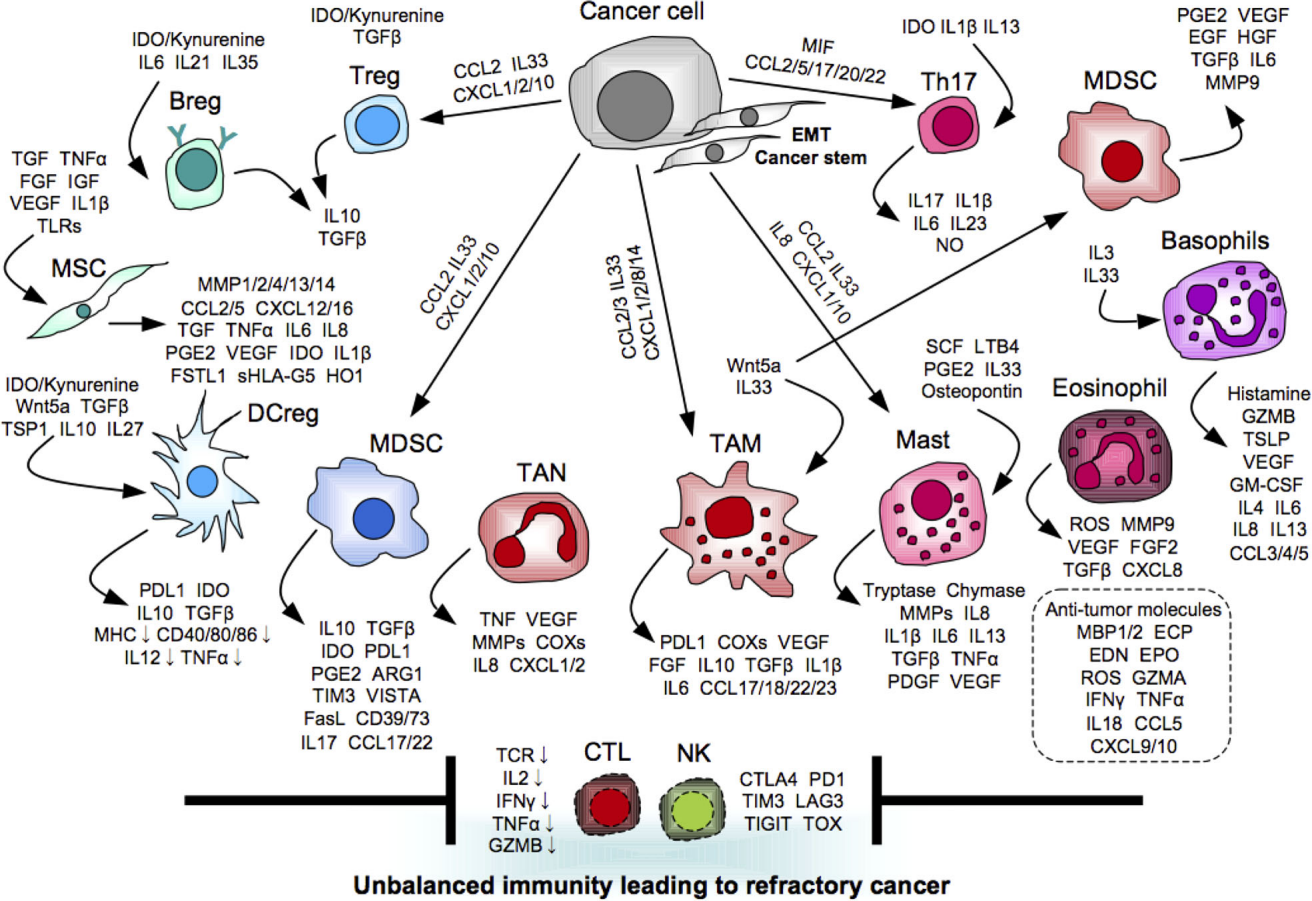
Myeloid orchestration leading to refractory cancer. Myeloid cells promote tumor progression and metastasis directly and indirectly *via* providing unbalanced immunity mediated by immunosuppressive and pro-inflammatory molecules to interfere induction and activation of anti-tumor effector cells.

The EMT inducers not only confer aggressive properties on tumor cells, but also create an immune tolerant environment for the successful escape. For example, transforming growth factor-β (TGFβ) stands out as a master regulator of the mechanisms. The canonical TGFβ-SMAD pathway plays a key role in the EMT program in cooperation with other signaling pathways, such as PI3K/AKT, ERK/MAPK, RHOA, and ROCK (87). Alternatively, TGFβ also suppresses cytotoxic functions of CTLs and NK cells directly by reducing the expression of perforin, granzyme B, and NKG2D in these cells, and also indirectly by inducing immunosuppression mediated by regulatory T cells (Tregs) and immature APCs (88). Another key regulator is Wnt5a that is a prototypical activator of the non-canonical Wnt pathway associated with the ROR1/AKT/p65 pathway (89). Wnt5 activates various EMT-governing transcription factors, including the SNAIL family SNAI1 (Snail) and the basic helix-loop-helix factor TWIST, and consequently induces downregulation of adhesion molecules including occludin, ZO1/2, and E-cadherin, but upregulation of mesenchymal molecules including β-catenin, N-cadherin, vimentin, and fibronectin (85). Alternatively, Wnt5a stimulates Møs to secrete immunosuppressive molecule IL10 through the toll-like receptor (TLR)/MyD88/p50 pathway followed by suppression of DC maturation (89). The EMT-undergoing tumor cells further disturb induction and activation of anti-tumor immune responses by orchestrating immunosuppressive and pro-inflammatory cells to build up tolerant and supportive environment for raising the probability of its successful escape (**Figure 1**). We next summarize the molecular and cellular mechanisms underlying the oncoimmunological network, especially mediated by myeloid cells, which are the major component in the human immune system.

### 3.1 Immunosuppressors for tumor escape

Snail is an EMT-governing transcription factor. Snail^+^ tumor cells produce thrombospondin-1 (TSP1) to promote tumor EMT in an autocrine manner, and indirectly through the generation of Treg-inducible regulatory DCs (DCreg) (90). CD47 is a receptor for TSP1, and the significant relationship between its high level and poor prognosis in various types of cancer, including GI cancer. For example, CD47 protein is aberrantly expressed in tumor tissues of GC patients, and the positivity is significantly associated with resistance to fluorouracil-based adjuvant chemotherapy, and the consequent poor prognosis (91). This study also showed that CD47 mRNA expression is especially enriched in GC with MSI and ARID1A mutation. The snail^+^ tumor cells also produce follistatin-like 1 (FSTL1) to promote tumor EMT in an autocrine manner, and indirectly through the induction of immune exhaustion and dysfunction, and apoptosis in CTLs (92–94). TP53 abnormality (loss, decrease, inactivation, mutation) generates cancer stem cells (CSCs) through the EMT signaling, and induces production of various chemokines, such as CCL2, CXCL1/2, and CXCL10, to recruit immunosuppressive cells, including Tregs and MDSCs (95). CSCs produce a cytosolic heme-containing enzyme indoleamine 2,3-dioxygenase (IDO) that degrades tryptophan into kynurenine followed by activation of AhR and GCN2 in immune cells (96). Tryptophan is essential for maintaining physiological and immunological homeostasis. The kynurenine-AhR/GCN2 axis widely suppresses cytotoxicity, proliferation, and survival of T cells and NK cells directly, and also indirectly *via* generating various immunosuppressive cells, such as Tregs, regulatory B cells (Bregs), DCregs, and MDSCs (96). IDO also regulates tumor dormancy that is a hallmark of CSCs by triggering G0/G1 cell cycle arrest (96).

Tregs are a heterogeneous population expressing tissue- or function-specific transcription factors, such as GATA3 and STAT3, along with FOXP3 that is a hallmark transcription factor of Tregs, and are the most prominent immunosuppressor that maintain self-tolerance and homeostasis as reviewed elsewhere (97). Bregs are generated *via* suppression of differentiation and maturation of B cells, and/or stimulation with pro-inflammatory cytokines, such as IL6, IL21, and IL35 (98). Bregs highly express and produce immunosuppressive and tumor-promotive molecules, such as PDL1, IL10, and TGFβ, as reviewed elsewhere (98). Here, we highlight immunosuppressive myeloid subsets, including MDSCs, DCregs, and MSCs.

#### 3.1.1 MDSCs

MDSCs are composed of mononuclear (M-MDSCs) and polymorphonuclear myeloid cells (PMN-MDSCs), and an immunosuppressive subset is defined by several markers, such as CD11b, CD14, Ly6C, Ly6G, MHC II, and CD33, in combination, since no specific single marker remains to be identified. MDSCs are expanded and activated particularly by hypoxia in the tumor microenvironment (99). Under the hypoxic condition, hypoxia-inducible factor 1-alpha (HIF1α) induces ectonucleotidases, CD39 and CD73, to transform into MDSCs, and these molecules convert ATP to adenosine that inhibits T-cell functions through the adenosine receptors (100). MDSCs produce various immunomodulatory molecules, such as TGFβ, IL10, IDO, prostaglandin E2 (PGE2), and ARG1, and highly express PDL1 and Galectin-9 that binds T-cell immunoglobulin mucin 3 (TIM3), followed by induction of steady immunosuppression (101). V-domain Ig suppressor of T-cell activation (VISTA) is also upregulated in MDSCs under hypoxic condition, and plays immunosuppressive roles like PDL1 (102). Immunosuppressive Møs called “type 2 Møs (M2-Møs)”are likely a part of MDSCs, since M2-Møs show immunosuppressive activities similar to those of MDSCs. For example, M2-Møs suppress CTL functions not only directly utilizing PDL1 and immunosuppressive cytokines, such as IL10 and TGFβ, but also indirectly *via* production of immunosuppressive cytokines, recruitment of Tregs by CCL23, and polarization of Th2 by CCL17, CCL18 and CCL22 (103).

Increase of MDSCs are strongly associated with accumulation of Tregs in the tumor tissues, probably because MDSCs can expand Tregs directly *via* CD40 expressed on the MDSCs (104), and also indirectly by recruiting Tregs into the tumor milieu *via* producing IL17. The MDSC-derived IL17 induces own production of CCL17 and CCL22 in an autocrine manner, and enhances immunosuppressive activity of the recruited Tregs by upregulating CD39 and CD73 (105). In clinical settings, the high frequency of MDSCs in tumor tissues and peripheral blood is significantly associated with tumor metastasis, higher stages, and poorer prognosis in GC (106, 107), CRC (12), or EC (108–110), suggesting a critical biomarker and possible target in the treatment of GI cancer. In GC, the high levels of M-MDSCs in peripheral blood (106) or PMN-MDSCs in tumor tissues (107) are significantly associated with poor prognosis of patients. The tumor-derived PMN-MDSCs have been shown to highly produce S100A8/A9, which promotes tumor progression directly by upregulating CXCL1 in tumor cells *via* the TLR4/p38-MAPK/NFκB pathway, and also indirectly by suppressing glycolysis, proliferation and tumor necrosis factor alpha (TNFα) and interferon gamma (IFNγ) production of CD8^+^ T cells *via* the TLR4/AKT/mTOR pathway, leading to anti-PD1 resistance (107). Also in EC, the PMN-MDSCs have been demonstrated as a predominant myeloid subset in tumor tissues, and the high levels of PMN-MDSCs are significantly associated with advanced staging, low grade, lymph node metastasis, HER2^-^ status, and poor prognosis of patients (108). M2-Møs has been also noticed in ESCC. For example, infiltration and polarization of M2-Møs are promoted by tumor-derived S100A7, which can directly promote tumor proliferation and migration *via* intracellular binding to JAB1 and paracrine interaction with RAGE receptors (109). This study also showed that the S100A7 positivity in tumor tissues is a poor prognostic factor. Interestingly, a pro-inflammatory cytokine IL32 is highly expressed in ESCC tumor tissues, and the IL32 derived from ESCC extracellular vesicles plays a key role in promoting lung metastasis by inducing M2-Mø polarization *via* the FAK-STAT3 pathway (110).

#### 3.1.2 DCregs

DCregs, alternatively called tolerogenic DCs, are a heterogeneous population. As no specific single marker has been identified, an immunosuppressive subset is defined by upregulation of immunosuppressive molecules (PDL1, IL10, TGFβ, and IDO), but downregulation of MHC II, T-cell co-stimulatory molecules (CD40, CD80, CD86, etc.), and pro-inflammatory cytokines (IL12, TNFα, etc) (111). However, the *in vivo* functions of DCregs, particularly in human, remain unclear. A possible reason is that the number of DCs is small and limited in a body, and DCregs are needed to be induced and be expanded for the analysis by the *in vitro* long-term culture that may modify the phenotypes. In the *in vitro* setting, DCregs can be generated *via* the tolerogenic signaling mediated by STAT3, AhR and SOCS2 in response to various stimuli, such as IL10, TGFβ, vitamin D3, and/or dexamethasone (112, 113). A pleiotropic cytokine IL27 also generates DCregs accompanied by CD39 upregulation *via* the STAT1/3 signaling (114). Interestingly, DCregs can be generated by stimulation with Helicobacter pylori that is a major cause of GC (115). In GC, DCregs expressing a non-classical and tolerogenic molecule HLA, HLA-G, significantly increase in peripheral blood of patients, and the high levels are significantly correlated with tumor grade, suggesting a critical biomarker in GC (116). HLA-G is also known as a poor prognostic marker in CRC (117). In CRC, tumor cells have been demonstrated to frequently suppress DC maturation, and generate immunosuppressive DCregs and dysfunctional DCs (118, 119). In ESCC, DCregs have been reported as a predominant subset in immune-suppressive cell populations within tumor tissues of patients using single-cell RNA sequencing, albeit few reports showing DCregs in EC so far (120).

#### 3.1.3 MSCs

MSCs with a broad tissue distribution are able to differentiate into a variety of mesenchymal lineages, such as adipocytes, osteocytes, chondrocytes, fibroblasts, and pericytes, suggesting the great and wide impact on the physiological conditions of the host (121). MSCs have been considered as a key player in tumor progression and metastasis leading to treatment resistance in GI cancer (122). As no specific single marker has been identified, human MSCs have been defined using several molecules, such as CD49a, CD73, CD90, CD105, CD146, CD271, and STRO1, in combination with negative expression of CD11b, CD14, CD19, CD34, CD45, CD79a (123). MSCs highly express various chemokine receptors, such as CCR2, CCR3, CXCR4 and CXCR5, and various metalloproteinases, such as MMP1/2/4/13/14 and tissue inhibitors of metalloproteinases (TIMP1/2), and thus promptly migrate into tumor sites in response to chemokines, such as CCL2, RANTES/CCL5, CXCL12, and CXCL16, within the tumor milieu (124). The migrated MSCs are expanded by cytokines, such as TGFβ, vascular endothelial growth factor (VEGF), fibroblast growth factor (FGF), and insulin-like growth factor (IGF). MSCs acquire immunosuppressive and pro-inflammatory properties upon the activation with the microenvironmental cytokines, such as TNFα and IL1β, and/or ligation of the TLRs, such as TLR2, TLR3, and TLR4 (123). The activated MSCs become to promote tumor progression and metastasis directly and indirectly through creating immune tolerant environment by producing numerous immunomodulatory molecules, such as TGFβ, PGE2, VEGF, TNFα, IDO, IL1β, IL6, FSTL1, HO1, and soluble HLA-G5 (125).

However, the *in vivo* functions of MSCs remain obscure despite the numerous studies in the world. As well as DCregs, the number of MSCs are extremely limited in a body, and MSCs are needed to be expanded for the analysis by the *in vitro* long-term culture. In addition, the sources of the MSCs vary depending on the studies. Furthermore, the phenotypes and biological characteristics of MSCs have been demonstrated in regenerative research without cancer. Cancer-associated MSCs must be different from the original MSCs brought up in the absence of cancer. Identification of the precise MSCs in patients with cancer is emergently needed for the practical application of targeting MSCs in cancer therapy.

In clinical settings, cancer-associated fibroblasts (CAFs) rather than MSCs have attracted greater attention as a predominant stromal subset in GI cancer. In CRC, CAFs produce M-CSF that stimulates CD163^+^ Møs to produce CCL2, HGF, IL6, and CXCL8/IL8 for recruitment and differentiation of monocytes into immunosuppressive Møs like M2-Møs in normal colon, potentially leading to tumorigenesis (126). Single-cell and spatial analysis of CRC tumor tissues also revealed the close relationship between FAP^+^ fibroblasts and SPP1^+^ Møs, and the positivity of both molecules are predictive of less therapeutic benefit from an anti-PDL1 therapy (127). In ESCC, CAFs generate M-MDSCs by the secreted IL6 and exosomal microRNA-21, and the CAF-induced M-MDSCs confer chemoresistance on tumor cells (128). The high levels of CAFs and CD11b^+^ M-MDSC-like cells are significantly associated with poor prognosis in ESCC.

### 3.2 Inflammatory facilitators for tumor escape

Persistent and strong stimulation with pro-inflammatory mediators seriously damages the immune system, and facilitate tumor development, progression, and metastasis, leading to treatment failure. Myeloid cells produce a variety of pro-inflammatory molecules, such as cyclooxygenases (COXs), prostanoids, arginase 1, TNFα, IL1β, IL4, IL6, IL10 and IL13, and greatly affect multiple steps of tumor evolution, including genomic instability, metabolic reprograming, stromagenesis, angiogenesis, invasion, dissemination, and modification of host immunity (129). Th17 cells also participate in the inflammatory process for tumor progression and metastasis, albeit partly paradoxical depending on the study condition. Th17 cells are generated by tumor-derived IL1β and IL13, and accumulate in tumor tissues in response to various chemokines, including CCL2, CCL5, CCL20, CCL17, CCL22, and MIF, which are produced from tumor cells (130). Th17 cells highly produce pro-inflammatory molecules, such as IL17, IL1β, IL6, IL23, and nitric oxide (NO), and promote tumor progression directly, and also indirectly *via* inducing angiogenesis. Interestingly, Tregs are converted into Th17 cells by IDO stimulation in tumor-draining lymph nodes (131).

The chronic inflammation induces immune exhaustion and dysfunction by firmly braking the immune activation signals *via* inducing expression of multiple IC molecules, including CTLA4, PD1, TIM3, LAG3, and TIGIT, in anti-tumor effector cells (132, 133). Consequently, anti-tumor effector molecules, such as IL2, IFNγ, TNFα, and granzyme B (GZMB), is dramatically downregulated in the CTLs and NK cells, and immune exhaustion and dysfunction are provoked locally and systemically in the host. LAG3 suppresses anti-tumor immunity directly by TCR downregulation, and also indirectly by impeding CD4^+^ T-cell functions *via* competitively binding to MHC II with a higher affinity (134). TIGIT also suppresses anti-tumor immunity by TCR downregulation upon the binding to the ligands, CD155 (PVR) and CD112 (Nectin2), expressed in myeloid cells and tumor cells (135). Exhaustion and dysfunction of NK cells are fear in cancer immunotherapy, since CTLs sometimes miss tumor cells due to the MHC loss on tumor cells as described above. Recently, an HMG-box transcription factor, thymus high mobility group box protein (TOX), was identified as a key regulator of exhaustion of T cells (136). TOX expression is induced by calcineurin and NFAT2, and orchestrates immune inhibitory signals, not only PD1 but also other IC molecules, in CD8^+^ T cells (137). Interestingly, the TOX binding to PD1 promotes the endocytic recycling of PD1 to maintain abundant PD1 expression on the cell surface, and sustains exhausted status of T cells. CD101 was identified as a marker to distinguish transitionally exhausted T cells, which still exert anti-tumor activities by invigoration, from terminally exhausted and dysfunctional T cells (47).

Here, we summarize pro-inflammatory myeloid subsets, including neutrophils, Møs, mast cells, basophils, eosinophils, which negatively impact on induction of anti-tumor immunity.

#### 3.2.1 Neutrophils

Neutrophils are the most abundant cellular components in the human immune system. Tumor-associate neutrophils, called “TANs”, are generated by various cytokines within the tumor milieu, and become to produce a variety of cytokines, such as TNFα, VEGF, and MMPs, and chemokines, such as CXCL1, CXCL2, and CXCL8/IL8, for promoting tumor growth and metastasis, angiogenesis, inflammation, and immunosuppression (138). The significant association between the high levels of neutrophils and poor prognosis has been demonstrated in GC (139–141) and CRC (142). However, the results are sometimes inconsistent potentially due to the high heterogeneity, plasticity, lack of the specific markers, and the short lifespan followed by rapid turnover in the host.

In clinical settings, neutrophil-to-lymphocyte ratio (NLR) in peripheral blood has been noticed as a marker of a systemic inflammatory status in patients, particularly with GC. The elevated NLR is significantly correlated with distant tumor dissemination, such as lymph node metastasis, peritoneal metastasis, osseous metastasis, and hepatic metastasis in GC (139). The elevated NLR is also significantly associated with poor prognosis of AGC patients after anti-PD1 therapy (140). The high levels of CD66b^+^ TANs at the invasion margin have been reported as another poor prognostic marker in GC (141). This study showed that TANs promote tumor EMT by the secreted IL17a *via* the JAK2-STAT3 signaling pathway. Neutrophils form extracellular fibrous scaffolds constituted of its nuclear and cytoplasmic proteins, called “neutrophil extracellular traps (NETs)”, upon the activation, and the NETs have been shown as a pathogenic factor in GI diseases, including GI cancer (143). For example, NETs in peripheral blood and ascites fluids promote tumor extravasation and dissemination into liver and peritoneum leading to metastasis in GC (144).

#### 3.2.2 Møs

Møs with a longer lifespan than polymorphonuclear cells are the most outstanding player in the inflammatory responses linking to cancer progression and metastasis, and have attracted great attention as tumor-associated Møs (TAMs) in cancer. Pro-inflammatory TAMs are recruited by microenvironmental chemokines, such as CCL2, CCL3, CXCL1, CXCL2, CXCL8/IL8, and CXCL14, to tumor tissues, and produce pro-inflammatory and pro-angiogenic molecules, such as cyclooxygenases (COXs), IL1β, IL6, VEGF and FGF, for promoting tumor progression and metastasis in there (103). COXs produce eicosanoids such as prostaglandin E2 (PGE2) and thromboxane 2 (TXA2) from arachidonic acid to cause inflammation (145). COX1 is constitutively expressed in most tissues, but is upregulated in some types of cancer. In contrast, COX2 is induced by pathogenic stimuli not only in tumor cells, but also in other cells, such as fibroblasts, chondrocytes, endothelial cells, and Møs (146). IL1β enhances tumor invasion and dissemination directly, and also indirectly *via* inducing HIF1 expression followed by VEGF production (147). FGF synergizes to promote the VEGF-caused angiogenic process, including migration and proliferation of endothelial cells, and formation of transdifferentiated capillary tubes (148).

Pro-inflammatory properties of MDSCs have been also demonstrated, suggesting a part of the TAMs. MDSCs induce the EMT program by releasing various cytokines, such as PGE2, TGFβ, EGF, and HGF, and strengthen the tumor stemness using IL6 that activates STAT3 and NOTCH pathways (100). The CSCs induce expand and activate MDSCs, and the feedback loop brings up intractable tumors. MDSCs are recruited and activated by IL33, and produce VEGF, FGF, and MMP9 for inducing angiogenesis and tumor invasion in collaboration with other ST2^+^ cells, including Mø and mast cells (149). The activated MDSCs also promote T-cell differentiation into pro-inflammatory Th17 for facilitating the inflammatory process and consequently tumor progression and metastasis.

In clinical settings, the high level of CD206^+^ TAMs in tumor tissues has been shown as a significant poor prognostic marker in GC patients with liver metastasis (150). Single cell analysis of tumor tissues revealed that GC patients with increase of HS6ST2^+^ tumor cells and SERPINE1^+^ Møs show unfavorable prognoses (151). These molecules are known to promote tumor growth, adhesion, and migration. In CRC, increase of CD163^+^ TAMs at the invasive front in tumor tissues is significantly associated with poor prognosis of patients (152). This study also demonstrated that CRC-induced TAMs promote tumor migration and invasion by its secreted IL6 that inhibits expression of a tumor suppressor miR-506-3p followed by production of CCL2 to further recruit TAMs. Another study reported that IL6-prodicing TAMs confer chemoresistance on CRC tumor cells *via* the IL6R-STAT3 signaling pathway that inhibits expression of a tumor suppressor miR-204-5p (153). The CCL2-CCR2 axis is also important in ESCC. CCL2 upregulation and TAM increase are significantly observed in ESCC tumor tissues, and are significantly associated with poor prognosis (154).

#### 3.2.3 Mast cells

Mast cells have pre-formed secretory granules containing classical and non-classical pro-inflammatory molecules, such as histamines, tryptase, chymase, heparin, lysosomal enzymes, and pro-inflammatory cytokines, such as IL6, IL8, TNFα, VEGF, FGF2, and platelet-derived growth factor (PDGF) (155), and are widely known to play a central role in inflammatory pathogenesis, particularly of allergy and cancer (156). Mast cells are recruited by the microenvironmental chemokines, such as CCL2, CXCL1, CXCL8/IL8, and CXCL10, to tumor tissues, and are activated by pro-inflammatory cytokines, such as stem cell facto (SCF), IL33, PGE2, leukotriene B4, and osteopontin, in there (157). SCF stimulates mast cells to produce tryptase and chymase *via* the tyrosine kinase activation signaling of the c-kit receptor, followed by activation of the released MMPs that degrade extracellular matrix components and tissues. The activated mast cells also produce IL1β, IL6, IL8, IL13, TGFβ, TNFα, PDGF, and VEGF for promoting tumor growth and metastasis directly, and also indirectly by provoking angiogenesis and immune chaos (155).

In particular, release of IL33, a member of the IL1 family, from mast cells is a disaster in cancer. IL33 is also released from many other cells, such as endothelial cells, epithelial cells, fibroblasts, and cancer cells, upon cellular stress. IL33 recruits and activates its receptor ST2-expressing cells, including not only pro-inflammatory cells (mast cells, TAMs, basophils, eosinophils, etc.), but also immunosuppressive cells (Tregs, MDSCs, ILC2s, etc.) followed by angiogenesis, immune tolerance, and inflammation in the host (149). IL33 is upregulated in diverse types of cancers, particularly in GC and CRC, and the IL33-mast-TAM axis has been reported as a poor prognostic factor in GC patients (158). In GC, however, IL33 is expressed mainly in epithelial cells, and partly in CD11b^+^CD64^+^MHC II^+^CX3CR1^+^ Møs, but not in MCPT1/2^+^ mast cells (159).

Many other studies have demonstrated the significant correlations among the high level of mast cells, angiogenesis, and tumor progression in many cancers, including GC (160) and CRC (161), while the opposite and favorable results have been also reported in several cancers, including EC (162). This inconsistency may partly depend on the proportion of Treg cells and the interaction between mast cells and Treg cells in the host. Because Tregs suppress mast cell functions, such as differentiation, degranulation, IgE-mediated LTC4 production by immunosuppressive cytokines, such as IL10 and TGFβ, and the Treg/OX40-mast/OX40L axis (163, 164), and conversely mast cells confer pro-inflammatory property to immunosuppressive Tregs without losing T-cell-suppressive properties, and promote inflammatory responses (165). Interestingly, a current study using humanized mice (NGS mice transplanted with human CD34^+^ cells and autologous thymus grafts) has demonstrated that co-localization of mast cells and Tregs in IL33^+^ tumor tissues is significantly associated with resistance to anti-PD1 therapy (166). They also showed that depletion of mast cells improves anti-PD1 therapeutic efficacy in the tumor models.

#### 3.2.4 Basophils

Basophils have pre-formed secretory granules containing pro-inflammatory cytokines, such as IL4, IL6, IL13, TSLP, GM-CSF and VEGF, and chemokines, such as CCL3, CCL4, CCL5 and CXCL8/IL8, in addition to histamine and granzyme B, and are widely known as a key player in allergy and parasitic infection (167). IL3 and IL33 are potent activators of basophils, and stimulate to produce these molecules. Despite the small number (< 1%) in peripheral blood leukocytes, accumulating evidence suggests that basophils participate in cancer pathogenesis, since basophils are a major source of IL4 that induce Th2 and M2-TAM polarization, and also produce CCL5 to recruit TAMs and Treg cells (168).

In clinical settings, the significant correlation between basophils accumulation in tumor tissues and patient survival has been demonstrated in several types of cancers, including GC (169). Gene expression analysis of patient-derived GC tumors also showed that the high levels of basophil activation signatures (CD123, CCR3, FceRIa, CD63, CD203c, and tryptase) are significantly associated with poor prognosis, while the results are reversal in sarcoma and endometrial cancer (170). In CRC, basopenia (decrease of basophils) in peripheral blood is associated with poor prognosis (171), while the results are reversal in other cancers, including breast cancer and ovarian cancer (168). Thus, the significance of basophils is still controversial in cancer, and should be determined by the further investigations.

#### 3.2.5 Eosinophils

Eosinophils are widely known as a key player in allergy, parasitic and fungal infections, and asthma. However, eosinophils have only recently come to the fore in cancer, albeit still remaining inconsistency. For example, the high levels of eosinophils are significantly associated with poor prognosis in GC and CRC, but with better prognosis in lung cancer and ovary cancer (172). For the priming and expansion of eosinophils, IL5 is an essential molecule. IL5 stimulation induces expression of chemokine receptors for chemoattractant eotaxins (eotaxin1/CCL11, eotaxin2/CCL24, and eotaxin3/CCL26) and many other chemokines (CCL8, CCL7, CCL13, CCL5, CCL15, etc.) rich in tumor microenvironment (172, 173). Eosinophils highly express ST2, RAGE, and Toll-like receptor 4 (TLR4), and are activated by the ligands, including IL33.

Two different types of eosinophils have been reported (172, 173). One is a tumor-promotive type that produces inflammatory and angiogenic molecules (ROS, VEGF, FGF2, MMP9, IL8, etc.), which induce genetic instability, DNA damage, angiogenesis, and EMT of tumor cells. Another is an anti-tumor type that produces the unique acidophilic secondary granules composed of major basic protein 1 and 2 (MBP1, MBP2) and a matrix composed of eosinophil cationic protein (ECP), eosinophil-derived neurotoxin (EDN), and eosinophil peroxidase (EPO), and many immunomodulatory molecules (granzyme A, TNFα, IL18, IFNγ, CCL5, CXCL9, CXCL10, etc.). These molecules directly exert cytotoxity on tumor cells, and also induce anti-tumor immunity *via* polarization of M1-TAMs. In clinical settings, the latter anti-tumor type has been implicated in GI cancer, including GC, CRC, and EC, based on the gene expression in tumor tissues, whereas the functions remain to be defined (172, 173).

## 4 Myeloid subsets expressing IC molecules

**Figure 2:**
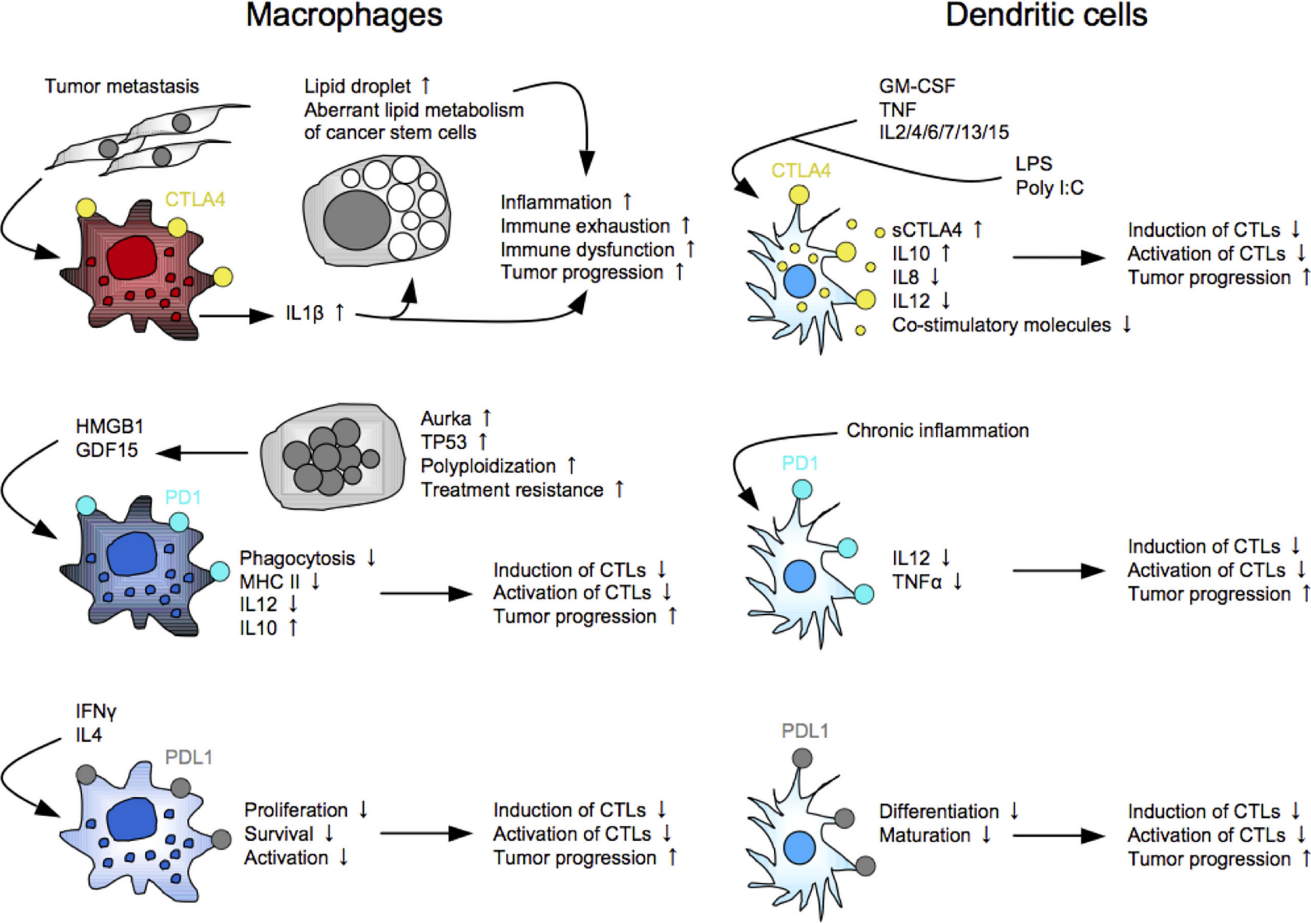
Myeloid subsets expressing immune checkpoint molecules. As well as PDL1, CTLA4 and PD1 are functionally expressed in myeloid cells, including macrophages and dendritic cells, and play key roles in induction of immune suppression and exhaustion in the host.

The general perception is that IC molecules, such as PD1, CTLA4, LAG3, and TIGIT, are expressed in T cells and NK cells, and the ligands are expressed in the other cells, including tumor cells and myeloid cells. Indeed, there are many reports showing the functions of the ligands expressed in tumor cells and myeloid cells. For example, PDL1 expressed in tumor cells functionally regulates cell proliferation and survival through the ERK/mTOR pathway (174), EMT induction through the RAS/ERK pathway (86), and cell metabolism through the Akt/mTOR pathway (175) in addition to the immune brake in the PD1-expressing cells. PDL1 expressed in myeloid cells induces Treg-inducible DCregs (176), and also suppresses Mø functions, such as proliferation, survival, and activation (177). However, accumulating evidence suggests the significant expressions and functional roles of the IC molecules in myeloid lineages (**Figure 2**). Most studies demonstrated DCs and Møs expressing expression of CTLA4 or PD1, but several studies reported unique subsets: a MSC subset expressing membrane-bound and soluble CTLA4 that is responsible for the immunosuppressive property (178), and a LAG3^+^CD11b^+^ myeloid subset that induce apoptosis in CTLs (93). This fact opens up new possibilities of indication expansion of ICIs for targeting myeloid cells, which exist and increase much more than T cells and NK cells in cancer patients, while the clinical relevancy of targeting these cells remain to be determined.

### 4.1 CTLA4^+^ myeloid subsets

The first report demonstrated CTLA4 expression in human monocytes and myelomonocytic cell lines U937 and THP1 upon the activation with PMA and IFNγ (179). This study also showed that blocking the myeloid CTLA4 partially inhibits its proliferation and T-cell stimulatory molecule expression (CD86, CD54, HLA-DR and HLA-DQ) through the AP1-NFκB signaling pathway. CTLA4 is expressed in monocytes after differentiation. For example, bone marrow monocyte-derived DCs express membrane-bound and soluble CTLA4 upon the maturation with LPS, Poly I:C or inflammatory cytokines, and the CTLA4 ligation with an agonistic anti-CTLA4 mAb enhances IL10 production but suppresses IL8, IL12 and T-cell stimulatory activity (180, 181). The CTLA4 seems to brake the full maturation/activation of DCs. Interestingly, intracellular CTLA4 molecules are packaged in microvesicles of mature DCs, and the microvesicles are transferred to the neighboring DCs for suppressing maturation, suggesting a contagious brake in DCs (182). CTLA4^+^ TAMs are systemically expanded in mouse and human CRC metastatic settings, and facilitate tumor progression and metastasis directly by generating lipid droplets in tumor cells, and also indirectly by inducing immune exhaustion, leading to anti-PD1 resistance (183). Lipid droplets have been considered as a cellular organelle just for fat storage so far. However, accumulating evidence suggests its important roles in the aberrant lipid metabolism of tumor cells, and the increase of lipid droplets is now gathering attention as cancer stemness (184). Anti-CTLA4 therapy may contribute to alleviation of the inflammatory responses in CRC patients with increased CTLA4^+^ TAMs.

### 4.2 PD1^+^ myeloid subsets

A little later than the CTLA4 discovery, PD1 expression in myeloid cells has been demonstrated. DCs derived from PD1-knockout mice highly produce IL12 and TNFα, which are important for inducing potent CTLs, suggesting an immune brake role of PD1 in DCs (185, 186). PD1^+^ TAMs highly express CD206 and IL10, but not HLA-DR, CD64 and IL12, and suppress proliferation of CD8^+^ T cells (187). This study also showed that PD1^+^ TAMs are clonally expanded by exosomal HMGB1 derived from EC cells. PD1 ligation is a key component to suppress its phagocytosis of the PD1^+^ TAMs (188). Interestingly, PD1 is also expressed in Mø in the peritoneal cavity of mice and human. Ozawa et al. reported that PD1^+^ TAMs with dysfunctional phagocytosis are expanded in the peritoneal cavity with disseminated tumor cells in mouse CRC ascites models and GC patients (189). The peritoneal tumor cells are polyploidy (giant with large nuclei) highly expressing aurora kinase A (AURKA) and GDF15 that is partly involved in the PD1^+^ TAM expansion. They also showed that treatment with an AURKA inhibitor MLN8237 significantly induced anti-tumor immunity in the anti-PD1-resistant CRC ascites models, providing significant better prognosis. Peritoneal tumor dissemination is frequently seen in GI cancer, and leads to malignant ascites that suddenly and repeatedly relapses even after being drained from the peritoneal cavity, resulting in poor prognosis (190). Despite advances in molecular profiling of the intraperitoneal tumors and immune cells, and many clinical trials using inventive methods, such as cytoreductive surgery and hyperthermic intraperitoneal chemotherapy, therapeutic options for such patients are still extremely limited to palliative treatments of the symptoms (191). These findings may be a ray of light leading to improvement of the present status in the clinical settings. More clinical evidence of PD1^+^ myeloid cells has been demonstrated. For example, PD1^+^ DCs increase in tumor tissues and peripheral blood of patients with hepatocellular carcinoma (186), and the high levels of PD1^+^ TAMs in tumor tissues are significantly associated with poor prognosis in GC (192).

## 5 Treatment strategy for overcoming the ICI resistance

A promising strategy for successfully treating cancer is breaking the tumor-host interplay for impeding the reciprocal evolution producing oncoimmunological heterogeneity and complexity. Numerous agents, including small molecule inhibitors, antibodies, and genetically modified cells, have been clinically developed for treating cancer, but most clinical evaluations are still underway (7, 193). Targeting immune mediators is the most reasonable approach in cancer immunotherapy. Therefore, in addition to treatment regimens described in the clinical section, we summarize immunotherapeutics, which are likely to optimize the combination strategy for improving the clinical effectiveness of the ICI therapy, regardless of cancer types.

### 5.1 Targeting immunosuppressive molecules

As described repeatedly, the clinical efficacies of ICIs targeting CTLA4, PD1, and PDL1 are low in most cases, and thus many inhibitory mAbs targeting other IC molecules, such as TIM3 (TSR-022, MGB-453, INCAGN02390, Sym023, and BGB-A425), LAG3 (Relatlimab, LAG525, REGN3767, MK-4280, Syn-022, and TRS-003), and TIGIT (tiragolumab, BMS-986207, MK-7684, AB154, ASP8374, and COM902), have been pharmaceutically developed. These mAbs have been evaluated in combination with/without other agents, such as chemotherapeutics, molecular targeting inhibitors, and other ICIs, in numerous clinical trials. Bispecific mAbs that simultaneously inhibit two molecular pathways, such as PD1-TIM3 (RO7121661), PD1-LAG3 (RO7247669), and PDL1-LAG3 (FS118), have been also developed, and have been clinically evaluated for advanced and/or metastatic solid tumors, including EC. Anti-TGFβ mAbs (SAR-439459, NIS-793 and fresolimumab), and a small molecule inhibitor of TGFβ receptor I (TGFβRI) kinase for SMAD2 phosphorylation (galunisertib/LY2157299) have been clinically evaluated in combination with anti-PD1/PDL1 therapy in phase I/II trials for advanced solid tumors. M7824 is a bifunctional anti-PDL1-TGFβ trap fusion protein that not only reverts the mesenchymalization of tumor cells, but also activates CTLs and NK cells, and has been clinically evaluated in many trials for advanced solid tumors. Inhibitors targeting IDO1 (epacadostat, GDC-0919, PF-06840003, NLG802, SHR9146, and linrodostat), IDO2 (indoximod), or both (1-MT) have been clinically evaluated in combination with chemotherapy and/or ICI therapy in phase I/II studies for solid tumors and peritoneal cancer. In the ECHO-301/KEYNOTE-252 phase III trial, however, combination of epacadostat plus pembrolizumab showed no synergistic survival benefit as compared to the pembrolizumab monotherapy in patients with unresectable or metastatic melanoma.

Some of the ICIs directly affect myeloid villains expressing the IC molecules described above. However, most of these agents targeting immunosuppressive molecules do not directly affect immunosuppressive myeloid cells. Recently, however, several unique agents targeting immunosuppressive myeloid villains have been clinically developed. For example, anti-VISTA mAbs, including HMBD-002 (NCT05082610) and CI-8993 (NCT04475523), have been evaluated in combination with/without anti-PD1 therapy in phase I trials for advanced solid tumors, as VISTA is a marker of MDSCs, and also plays immunosuppressive roles. Anti-HLA-G mAb TTX-080 has been also evaluated in combination with/without pembrolizumab or cetuximab in phase I trials for advanced solid tumors, including CRC (NCT04485013), as HLA-G is a marker of DCregs and MSCs, and also plays immunosuppressive roles.

### 5.2 Targeting pro-inflammatory molecules

Basically, inflammatory mediators have been primarily targeted for treating other inflammatory diseases, such as rheumatoid arthritis and pulmonary disease, so far. However, several inhibitory mAbs targeting IL1β (canakinumab), IL6 (tocilizumab, siltuximab, etc.), and IL8 (BMS-986253) have been recently evaluated in combination with/without other agents, such as chemotherapy, anti-HER2 mAb, or anti-PD1 mAb, in many clinical trials for various types of cancers.

COXs are representative of pro-inflammatory molecules in tumor progression mechanisms, and there are a number of *in vivo* therapeutic studies showing the anti-tumor efficacies of a COX1/2 inhibitor aspirin in mouse tumor models (194). The reason may be that aspirin widely suppresses platelet aggregation, endothelial activation, tumor adhesion to the endothelium, recruitment of myeloid cells, and EMT induction in tumor cells. Also, the significant impact of aspirin use has been demonstrated in PDL1^low^ CRC tumors in clinical settings (195). However, aspirin therapeutic efficacy remains to be determined, since most of the clinical studies are retrospective, and COX2-specific inhibitor celecoxib is preferred in clinical therapy. Because COX1 is constitutive expressed in most tissues, whereas COX2 is inducible in pathogenic process, suggesting induction of adverse events by blocking COX1. Blocking COX2, however, may promote tumor metastasis *via* amplifying the COX1-induced events, since it has been shown that COX2 knockout upregulates COX1 that produces TXA2, which induces platelet aggregation to promote cancer metastasis, in mice (145).

### 5.3 Active immunotherapy

Induction and activation of anti-tumor immune responses is a fundamental strategy in cancer immunotherapy, and thus many immunomodulatory agents, including whole tumor vaccines, DC vaccines, tumor antigen peptides, and viral vectors, have been clinically developed so far, while most clinical trials have failed. Of note, tumor antigens have been recently re-focused as “neoantigens” based on the concept that higher mutations in tumor cells could lead to high immunogenicity that can induces immune responses (196). Numerous neoantigens have been identified using next generation sequencing and advanced bioinformatics technology, and various peptide vaccines (KRAS, DNAJB1-PRKACA, IDH1R132H, AE37, K27M, etc.) and the peptide-pulsed DCs have been clinically evaluated in combination with other treatments, such as chemotherapy and ICI therapy (197). Despite the great expectation, however, most trials have been failing. A potential reason may be the immunological diversity and complexity that can no longer be easily reprogrammed and fixed by the therapy.

### 5.4 Cell therapy

To elementally raise anti-tumor immunity, genetically engineered T cells and NK cells have been pharmaceutically developed for treating cancer as described previously (198). A great advantage of the CAR-NK therapy is that CAR-NK can be generated using not only autologous but also allogeneic donor cells, whereas only autologous T cells for CAR-T products. However, ex vivo expansion of NK cells is relatively difficult because the lifespan (< 10 days) is shorter than that of T cells (> 10 years) even in normal conditions. NKG2D-transduced CTLs has been recently developed, since NKG2D signaling activates anti-tumor effector cells *via* binding to the ligands (MICA/MICB, ULBP, RAE1, etc.) that are frequently overexpressed in tumor cells. NKG2D-CAR-T cells (CYAD-101, KD-025, NKX101, and NKR-2) have been now clinically evaluated in combination with chemotherapy in phase I/II trials for relapsed or refractory solid tumors (NCT03692429 and NCT04550663).

Three CAR-T products (tisagenlecleucel, axicabtagene ciloleucel, and brexucabtagene autoleucel) have been clinically approved for treating lymphoma, and one CAR-NK product (CellProtect) has been recently approved as an orphan drug for treating multiple myeloma. Despite the success in the treatment of hematological malignancies, however, the therapeutic efficacy is extremely limited in the treatment of solid tumors, and other issues, including serious adverse events, high manufacturing costs needed for the specialized facilities, and a few providers, remain to be solved in the clinical settings. Further improvement of the CAR design is needed for the successful treatment of solid tumors, including GI cancer.

## 6 Conclusions

Great advances in the profiling of genomic, proteomic, microenvironmental, and immunological approaches have been increasingly clarifying the oncoimmunological landscape underlying the resistance to ICI therapy, and different ICIs targeting other IC pathways and anti-cancer agents targeting multiple signaling pathways have been clinically developed. However, anti-tumor immune responses are not always induced and do not last long in all patients, and a significant proportion of patients acquire resistance to the treatment, possibly because of the oncoimmunological diversity and complexity. Disruption of the reciprocal evolution may successfully repel such refractory cancer. A promising strategy may be elimination and reprogramming of the myeloid villains that are the majority of cellular components in the human immune system. However, a single/dominant marker of the tumor-supportive subset should be identified, and the clinical relevancy of targeting the villain subset should be determined for the practical implementation of targeting myeloid cells in cancer therapy. This will greatly contribute to improvement of clinical outcomes, particularly in the ICI therapy of GI cancer.

## Author contributions

CK-S conceptualized, organized the draft manuscript, and wrote the manuscript. NB, HH, and HS drafted the manuscript. All authors contributed to the article and approved the submitted version.

## Conflict of interest

The authors declare that the work was conducted in the absence of any commercial or financial relationships that could be construed as a potential conflict of interest.

## Publisher’s note

All claims expressed in this article are solely those of the authors and do not necessarily represent those of their affiliated organizations, or those of the publisher, the editors and the reviewers. Any product that may be evaluated in this article, or claim that may be made by its manufacturer, is not guaranteed or endorsed by the publisher.
